# Natural Language Processing of Unstructured Healthcare Data for Predicting Heart Failure in Individuals with Type 2 Diabetes

**DOI:** 10.3390/jcm15093287

**Published:** 2026-04-25

**Authors:** Juan F. Navarro-González, Leopoldo Pérez de Isla, Gloria Cánovas Molina, Miguel Ángel Brito-Sanfiel, David Emilio Barajas Galindo, Luis Ángel Cuellar Olmedo, Dídac Mauricio, Santiago Tofé Povedano, José Antonio Balsa Barro, Matilde Rubio Almanza, José Juan Aparicio Sánchez, Miren Sequera Mutiozabal, Belén Pimentel, Ana Pérez Domínguez, Víctor Latorre Garrido, Claudia Maté, Daniel Salvador, Juan Francisco Merino-Torres, Antonio Jesús Blanco-Carrasco

**Affiliations:** 1 Unidad de Investigación y Servicio de Nefrología, Hospital Universitario Nuestra Señora de Candelaria, 38010 Santa Cruz de Tenerife, Spain; 2 RICORS2040 (RD24/0004/0022), Instituto de Salud Carlos III, 28029 Madrid, Spain; 3 Instituto de Tecnologías Biomédicas, Universidad de La Laguna, 38200 Santa Cruz de Tenerife, Spain; 4 Facultad de Ciencias de la Salud, Universidad Fernando Pessoa Canarias, 35450 Las Palmas de Gran Canaria, Spain; 5 Servicio de Cardiología, Hospital Clínico San Carlos, 28040 Madrid, Spain; 6 Servicio de Endocrinología y Nutrición, Hospital Universitario de Fuenlabrada, 28942 Madrid, Spain; 7 Servicio de Endocrinología y Nutrición, Hospital Universitario Puerta de Hierro, 28222 Madrid, Spain; 8 Servicio de Endocrinología y Nutrición, Hospital Universitario de León, 24080 León, Spain; 9 Servicio de Endocrinología y Nutrición, Hospital Universitario Río Hortega, 47012 Valladolid, Spain; 10 Servicio de Endocrinología y Nutrición, Hospital de la Santa Creu i Sant Pau, 08041 Barcelona, Spain; 11 Servicio de Endocrinología y Nutrición, Hospital Universitari Son Espases, 07120 Palma, Spain; 12 Servicio de Endocrinología y Nutrición, Hospital Universitario Infanta Sofía, 28702 Madrid, Spain; 13 Servicio de Endocrinología y Nutrición, Departamento de Medicina, Hospital Universitari i Politècnic La Fe, 46026 Valencia, Spain; 14 Instituto Investigación Sanitaria La Fe, Universidad de Valencia, 46026 Valencia, Spain; 15 Departamento Médico Cardiovascular, Renal y Metabolismo, AstraZeneca España, 28050 Madrid, Spainbelen.pimentel1@astrazeneca.com (B.P.);; 16 Departamento de Estrategia Digital e Innovación, AstraZeneca España, 28050 Madrid, Spain; 17 Medsavana S.L., 28004 Madrid, Spain; 18 Servicio de Endocrinología y Nutrición, Hospital Clínic de Barcelona, 08036 Barcelona, Spain

**Keywords:** type 2 diabetes mellitus, heart failure, predictive model, electronic health records, natural language processing, real-world data

## Abstract

**Background/Objectives**: Type 2 diabetes mellitus (T2DM) is a multisystemic disease with overlapping metabolic, renal, and cardiovascular effects. Within the Diabetic@ project, which aims to characterize individuals with T2DM using real-world data extracted from electronic health records (EHRs), this substudy sought to develop a predictive model for two-year heart failure (HF) risk. **Methods**: Multicenter, retrospective study including T2DM individuals across eight Spanish hospitals (2013–2018). Data were extracted exclusively from EHRs’ unstructured free text using clinical natural language processing (cNLP) and mapped to SNOMED CT. At inclusion, individuals were categorized as having or not prevalent HF (pHF). Predictive modeling was performed in non-pHF to assess two-year risk of developing HF, termed incident HF (iHF). Logistic regression (LR), decision trees, random forest, and XGBoost were compared, selecting for accuracy and interpretability. **Results**: Of 588,756 individuals with T2DM, 84,197 (14.3%) had pHF. Among non-pHF, 353,371 (60%) were used for model development (90.7% training, 9.3% validation). iHF occurred in 13.6% of the training set and 11.4% of the validation set. Ischemic heart disease was present in 16.2% overall, 37.9% in pHF, and 12.6% in non-pHF. Glycosylated hemoglobin data was rarely reported (<15%). LR achieved the best performance (AUC-ROC 0.73) using 27 predictors. Reduced 12- and clinically refined 9-predictor models performed similarly, with the latter implemented in a web-based tool. **Conclusions**: Unstructured data from EHRs enabled development of a two-year HF risk model for individuals with T2DM, underscoring the potential of cNLP for risk stratification across the cardiovascular–renal–metabolic spectrum.

## 1. Introduction

With a projected global burden of up to 629 million cases by 2045, type 2 diabetes mellitus (T2DM) represents a leading cause of morbidity [1]. T2DM is a multisystemic disease with overlapping metabolic, renal, and cardiovascular effects and significantly raises the risk of cardiovascular complications, especially heart failure (HF), a frequent and severe complication that is often underdiagnosed until advanced stages [2]. Approximately 30% of individuals with T2DM are likely to develop HF during their lifetime [3], and those with both conditions experience greater functional decline, higher hospitalization risk, more frequent readmissions, and increased cardiovascular and all-cause mortality, with higher healthcare costs [2,4]. The clinical interplay between T2DM and HF is complex and bidirectional: diabetes nearly doubles the lifetime risk of HF, while pre-existing HF contributes to insulin resistance and raises the incidence of new-onset diabetes [5,6,7,8].

Chronic exposure to cardiometabolic stressors characteristic of T2DM—such as hyperglycemia, hyperinsulinemia, dyslipidemia, low-grade inflammation, obesity, and persistent hypertension—progressively damages myocardial structure and function [9]. Over time, these mechanisms contribute to cardiac remodeling, left ventricular hypertrophy, and diastolic dysfunction, ultimately leading to clinical HF, with longer diabetes duration further increasing the risk [10,11]. As many individuals with T2DM exhibit subclinical structural or functional cardiac abnormalities years before symptoms appear [12], this early phase presents an opportunity for risk assessment, making predictive models a valuable tool for identifying high-risk individuals and guiding preventive strategies.

In recent years, several models have been proposed to predict HF onset or hospitalization in persons with T2DM. However, most of these models were developed using highly selected populations and a limited set of variables, which restricts their generalizability and applicability in real-world clinical settings [13,14,15,16]. In this context, electronic health records (EHRs) have emerged as a valuable source of clinical information for studying chronic diseases, as they provide longitudinal clinical data encompassing comorbidities, treatments, and subtle clinical indicators that may precede overt organ damage [17,18,19]. These data can help identify early risk patterns and support timely detection in routine clinical practice, yet much of this information remains embedded in unstructured free-text narratives.

Clinical natural language processing (cNLP) and machine learning (ML) are crucial for transforming unstructured clinical data into structured insights. Harnessing this often-underused data source enhances risk prediction, supports more informed clinical decisions, and offers opportunities for earlier disease detection and intervention [20]. Recent studies have highlighted the potential of NLP to improve disease understanding and outcomes in cardiology [21], including HF prediction [22]. Despite these advances, additional models developed in broader or more representative populations, particularly those reflective of local clinical practice, are still needed to ensure generalizability and clinical relevance.

This study is part of the Diabetic@ project, which explores the application of cNLP and ML to free text from EHRs to characterize individuals with diabetes. Previous publications from this project have described the hospital-attended population with T2DM in Spain [23] and developed a predictive model for chronic kidney disease (CKD) risk [19], demonstrating the feasibility of AI-driven risk prediction using unstructured real-world data (RWD). The present study aimed to develop and validate a predictive model for two-year HF risk in individuals with T2DM using the same underlying population and data source as previous Diabetic@ studies, relying exclusively on unstructured EHR data extracted from routine documentation across eight Spanish centers. This work addresses the need for scalable, real-world HF risk stratification tools that reflect the complexity and heterogeneity of everyday clinical practice, enabling earlier identification of high-risk individuals who may benefit from targeted preventive interventions.

## 2. Materials and Methods

### 2.1. Study Design and Study Population

This substudy is part of the Diabetic@ project, a multicenter, retrospective, observational study using RWD [23]. Clinical information was extracted from unstructured data from EHRs of persons with DM between 1 January 2013, and 31 December 2018. The study was conducted across eight tertiary referral hospitals representing five regions of the Spanish National Healthcare Network (Table S1). For this analysis, we included only adult individuals with T2DM attended in hospital settings, including both inpatient admissions and hospital outpatient visits, and excluded individuals under 18 years of age.

We conducted a descriptive and retrospective cross-sectional analysis at the inclusion date and during the follow-up period, defined as the time from inclusion to the last available EHR within the study period. To reconstruct the patient’s history, all available information before the inclusion date was analyzed. Additionally, we developed a predictive model for HF development. For the descriptive analysis, individuals with T2DM were classified as having prevalent HF (pHF), defined by mentions indicating a history of HF of any etiology occurring before the index date (T2DM diagnosis) (e.g., “HF since 2018” or “HF under treatment”), or as non-prevalent HF (non-pHF) at the inclusion date, defined as the earliest date on which a diagnosis of T2DM was recorded. For predictive modeling, only non-pHF individuals were considered and stratified into those who developed HF within a 2-year follow-up, termed incident HF (iHF), and those who did not, termed non-incident HF (non-iHF). iHF was identified when a new documented mention of HF appeared in the free-text EHR (e.g., ‘new-onset HF’, ‘signs or symptoms compatible with HF’, or ‘admission for HF’). In line with the real-world focus of the study, HF identification was based on routine clinical documentation, without requiring confirmatory diagnostic tests such as echocardiography or etiologic specification. Individuals who had less than 2 years of follow-up without developing HF were excluded from these analyses.

### 2.2. Study Variables and Data Extraction

Clinical experts defined and curated the variables included in the study, encompassing patient demographics, complications (i.e., diagnosed conditions rather than symptoms), laboratory parameters, comorbidities, and pharmacological treatments. For any variable analyzed at a particular time, the value closest to the time point within the reference time windows was used. These reference time windows were established to account for differences in clinical practice between individuals, healthcare providers, and hospitals, ensuring the greatest possible retrieval of relevant data from EHRs. The specific time windows for each variable or group of variables are provided in the table footnotes.

Clinical data recorded as unstructured free text and their context were extracted using EHRead^®^, Medsavana’s proprietary technology, which applies NLP and ML to convert free-text data into organized, standardized outputs using SNOMED CT terminology [24,25]. A predefined list of SNOMED CT terms representing the study variables, including common synonyms and acronyms used in clinical practice, was first created. The system automatically identified these entities within the clinical texts, mapped them to standardized SNOMED CT concepts, and enriched them with contextual attributes such as negation and temporality. Extracted entities, together with their associated information (e.g., clinical department, document type, and contextual attributes), were integrated into a synthetic database from which the study variables were constructed and analyzed [26]. EHRead^®^’s performance was validated as in previous studies [27], with results shown in Table S2. Additional details on the EHRead^®^ technology, including data sources, data processing, and extraction procedures, are available in previous Diabetic@ publications [19,23].

### 2.3. Descriptive Analysis

Baseline characteristics were defined as clinical entities documented before the index date, established as the date of T2DM diagnosis. Incident conditions were defined as those first identified during follow-up, provided no prior mention existed in the pre-index period. Categorical variables were summarized as frequencies, while continuous variables were described using mean, standard deviation (SD), median, and first and third quartiles (Q1, Q3). For continuous variables, the number of available values was also reported. Missing categorical data in free text were assumed to indicate absence of the feature. (i.e., actual zero values). The cumulative incidence of complications was estimated using the Kaplan–Meier method to account for censoring. Trends in laboratory parameters were assessed using generalized additive models (GAMs). Both Kaplan–Meier curves and GAM outputs were presented with 95% confidence intervals (CIs). Results were stratified by HF status at the time of inclusion. Data analyses were performed using R software (version 4.0.2) and Python (version 3.7.12).

### 2.4. Predictive Model Training and Validation

The predictive model was designed to estimate the risk of HF within two years of the inclusion date. It was trained using data from six hospitals and validated on data from two additional sites, with cohorts defined exclusively by geographic location—independent of clinical or demographic characteristics—to evaluate model generalizability to independent populations. Forty-two potential predictors, covering demographics, comorbidities, treatments, and laboratory parameters, were selected by medical experts based on clinical criteria and literature review (Table S3). Missing data from numerical variables was imputed using random forest multiple imputation, when required. Variables with more than 50% missing data were excluded to guarantee an adequate degree of completeness. This threshold was selected to support reliable estimation and to limit dependence on imputation, given that missingness was likely not at random, as clinicians tend to document abnormal values more frequently than normal ones in free-text notes. Under these conditions, imputing variables with extreme and systematically patterned missingness was considered likely to adversely affect model stability and the plausibility of imputed values.

All available predictors were used to train four ML models (decision tree, random forest, extreme gradient boosting [XGBoost], and logistic regression [LR]) in six hospitals, with default hyperparameters and a fixed random seed. Class imbalance was corrected using class-weight adjustments, assigning higher weights to the minority class to counteract its lower frequency. This approach increases the contribution of iHF cases during model training, enabling improved identification of underrepresented outcomes without oversampling or modifying the original data distribution.

Model performance was assessed with stratified 10-fold cross-validation. Alongside the ‘full model’, which included 27 predictors, we developed simplified versions. A 12-predictor ‘reduced model’ was generated using feature selection via the Maximum Relevance Minimum Redundancy (MRMR) method [28], while a 9-predictor ‘refined’ model was created through a review by multidisciplinary clinical panel from Spanish scientific societies of endocrinology, cardiology, and nephrology. During a series of workshops with HF specialists, the models were evaluated, adjusted, and finalized based on clinical judgment and consensus. The full, reduced, and refined models were subsequently validated on the two remaining hospitals, with performance assessed by area under the curve (AUC) receiver operating characteristic (ROC) and precision-recall curves, and confidence intervals (CIs) estimated by bootstrapping. For the selected model, the lift factor, defined as the ratio between model precision and the event prevalence in the validation cohort, was calculated to quantify the degree of risk enrichment achieved relative to random classification. For practical use, the most accurate and interpretable model was implemented in a user-friendly web application (Predict 2 Prevent, P2P), enabling researchers to input patient characteristics and obtain HF risk predictions. Supplementary Material provides further methodological details.

### 2.5. Ethical Considerations and Study Approval

This study was designated a non-post-authorization study by the Spanish Agency for Medicines and Health Products (AEMPS) and received approval from the Institutional Review Board at each participating site. All procedures and analyses adhered to applicable local regulations and legal requirements, as well as established research standards, as described in the latest Declaration of Helsinki and Good Pharmacoepidemiology Practices. Data were derived from anonymized EHRs that had been irreversibly dissociated, eliminating the need for individual patient consent. As a retrospective study using RWD, data collection and variable adjudication were conducted in a blind manner. All variables were automatically extracted from routine EHR documentation, with no additional data collection or study-specific clinical activities performed. Study reporting follows the Transparent Reporting of a multivariate predictive model for Individual Prognosis or Diagnosis (TRIPOD) guidelines [29].

## 3. Results

### 3.1. Study Population

**Figure 1:**
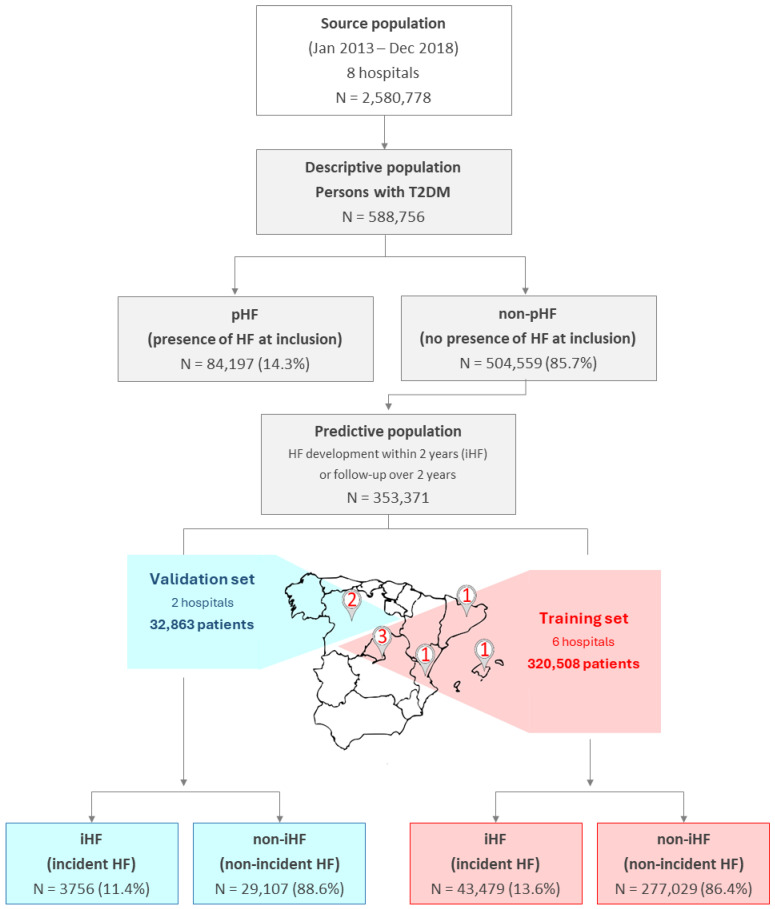
Population flow chart. Individuals included in the descriptive analysis (descriptive population, stratified by pHF and non-pHF at inclusion) and in the predictive analysis (predictive population, restricted to individuals with T2DM without HF at inclusion and excluding those with less than two years of follow-up without evidence of developing HF). The development of HF in the predictive population was termed incident HF (iHF), while individuals who did not develop HF during the follow-up were designated as non-iHF. Numbers on the map indicate the number of participating hospitals per region. Colored areas denote the geographic assignment of regions to either the training (red) or validation (blue) dataset. The training set (red) includes three hospitals from Madrid (Hospital Universitario de Fuenlabrada, Hospital Universitario Puerta de Hierro, and Hospital Universitario Infanta Sofía), one from the Balearic Islands (Hospital Son Espases), one from Valencia (Hospital Universitari i Politècnic La Fe), and one from Catalonia (Hospital de la Santa Creu i Sant Pau). The validation set (blue) includes two hospitals from Castilla y León (Hospital Universitario de León and Hospital Universitario Río Hortega de Valladolid). Abbreviations: T2DM: type 2 diabetes mellitus; HF: heart failure; pHF: presence of HF at inclusion; non-pHF: no presence of HF at inclusion; iHF: incident HF; non-iHF: non-incident HF.

During the study period, 2,580,778 individuals attended the participating hospitals, of whom 588,756 (22.8%) had a diagnosis of T2DM. Within this group, 84,197 individuals (14.3%) were classified as pHF (Figure 1). A total of 353,371 non-pHF individuals (60.0%) were included in the predictive model development after applying the exclusion criteria. The training set consisted of 320,508 individuals (90.7%), of whom 43,479 (13.6%) developed HF during the subsequent two-year follow-up (iHF). The validation set comprised 32,863 (9.3%) individuals, of whom 3756 (11.4%) were iHF (Figure 1).

### 3.2. Descriptive Analysis

The main characteristics and treatments of individuals included in the predictive analysis are shown in Table 1. In the training set, the median (Q1, Q3) age was 68 (55, 80) years for iHF individuals and 54 (39, 68) years for non-iHF individuals. Among those who developed HF, 49.8% were female. Dyslipidemia and CKD were reported in 55.8% and 60.5% of iHF individuals, and in 27.1% and 12.6% of non-iHF individuals, respectively. The use of antihypertensive treatment and statins was reported in 47.9% and 25.7% of individuals with iHF, and in 25.7% and 15.7% of those without iHF, respectively. These findings were consistent with the validation set.

Further details on demographic, clinical, and analytical characteristics at baseline of individuals with T2DM (overall, pHF, and non-pHF) are provided in Table S4. The median (Q1, Q3) age was 71 (56, 83) years for pHF individuals, and 56 (41, 71) years for non-pHF individuals. Overall, high blood pressure (HBP) (65.1%), dyslipidemia (59.5%), and CKD were commonly reported. While HBP and dyslipidemia were reported at similar rates in the pHF and non-pHF groups (around 60%), CKD was reported in 36% of pHF, but 16.4% of non-pHF. The cumulative incidence of major cardiovascular comorbidities and complications, as well as the evolution of laboratory variables in the pHF group are described in Figures S2 and S3, respectively.

### 3.3. Predictive Model Training and Validation

The four ML models were trained using 27 of the original 42 predictors; 15 laboratory parameters were discarded for having >50% missing values. Table 2 presents the performance metrics of the full, reduced, and refined models. Among the full models, XGBoost and LR performed best in the cross-validation, with mean (95% CI) AUC-ROC values of 0.74 (0.73–0.75) and 0.73 (0.72–0.75), respectively. Despite similar performance, the LR model was chosen because it offers better interpretability.

The reduced LR model with 12 predictors (CKD, loop diuretics, atrial fibrillation, cerebrovascular disease, ischemic heart disease, peripheral vascular disease, smoking, anticoagulants, antihypertensives, beta blocking agents, antiplatelet agents, and age at index) achieved an AUC-ROC of 0.73 (0.71–0.75) with variable importance shown in Table S5. Following a clinical panel review, the predictors potassium-sparing agents, diabetic retinopathy, and peripheral vascular disease were removed, while cerebrovascular disease and obesity were added. The final refined LR model included the nine predictors shown in Table S5 with their respective variable importances, yielding an AUC-ROC of 0.73 (0.72–0.75) (Figure S1). As all LR models showed similar performance, the refined model was chosen for implementation in the P2P risk prediction application, because of its curation and simplicity.

The full, reduced, and refined LR models were tested on the validation set, showing a mean (95% CI) AUC ROC and recall scores of 0.69 (0.68–0.70) and 0.79 (0.78–0.80), 0.68 (0.68–0.69) and 0.78 (0.77–0.79), and 0.68 (0.68–0.69) and 0.77 (0.76–0.79), respectively. In the validation cohort, the prevalence of iHF was 11.4%, and the selected model achieved a precision of 0.16 (95% CI: 0.16–0.16) (Table 2), corresponding to a lift factor of 1.4.

## 4. Discussion

In T2DM, early identification and comprehensive risk assessment of HF are crucial for optimal management, particularly in hospital settings where patients often present with advanced or unrecognized disease. However, HF remains frequently underdiagnosed, partly due to heterogeneous definitions and classification criteria, which complicates timely recognition and intervention [30]. To address this gap, our RWD study applied expert-refined cNLP and ML methods to extract clinical information from EHRs and develop a predictive model for HF onset in a large cohort of Spanish individuals with T2DM.

Our results revealed a high burden of HF among individuals with T2DM, with a prevalence of around 15%, and early development in a relevant percentage of cases (~14%). Individuals who developed HF also exhibited a high prevalence of other cardiovascular comorbidities, such as atrial fibrillation and ischemic heart disease, both well-established risk factors for HF. We also observed a strong relationship between HF and CKD, with CKD being both a coexisting comorbidity in pHF cases and a relevant predictor of HF onset. Consistent with this, we previously demonstrated within the Diabetic@ project that HF was a relevant predictor of CKD development [19]. There is solid evidence highlighting the close interconnection between T2DM, HF, and CKD [31], emphasizing the epidemiological, pathophysiological, and prognostic relevance of this triad. This has led to the adoption of the unifying concept of cardiovascular–metabolic–renal disease, now recognized as one of the most pressing public health challenges of the 21st century, underscoring the urgent need for integrated and proactive preventive strategies [2,11,31]. This well-established interplay further reinforces the importance of early identification and risk stratification in this high-risk population.

Building on this rationale, we developed ML-based predictive models for HF in individuals with T2DM, combining automated predictor selection with expert input. Our approach leverages cNLP to extract large datasets from free-text EHRs, capturing the richer, context-aware clinical information contained in narrative notes, where HF symptoms are often first documented. This highlights the added value of NLP in accessing nuanced data not typically available in structured fields, addressing key limitations of traditional models. In this regard, while previous HF prediction models in T2DM have demonstrated good discriminatory performance (AUC ~0.75–0.85), most rely solely on structured data and were developed in clinical trial cohorts, limiting their applicability to real-world populations [18]. Although one prior model incorporated NLP, it was not designed for a T2DM population, was based on a smaller dataset, and lacked independent validation [22]. To our knowledge, our model is the first to apply cNLP specifically to individuals with T2DM. It achieved moderate performance (AUC 0.69) and was refined for clinical interpretability, making it suitable for real-world hospital settings to support early identification and personalized management. Another strength of our study is the large volume of EHRs analyzed from eight Spanish hospitals, which were used to design the methodology and validate the predictive models, and enabled the creation of a clinically applicable model. This work extends the Diabetic@ framework by integrating HF alongside CKD prediction, laying the foundation for a scalable, multi-outcome risk stratification tool for individuals with T2DM. The development of a ready-to-use web-based tool further supports its potential for real-life implementation.

Several limitations should be acknowledged. First, iHF was identified based on clinician-documented mentions in free-text EHRs without requiring confirmatory diagnostic tests or etiologic specification. While this approach may reduce diagnostic specificity and introduce heterogeneity, it reflects real-world clinical practice and is consistent with the model’s intended role. As with any retrospective RWD analysis, residual confounding bias cannot be excluded despite we employed clinically guided feature selection and adjustment for key covariates whenever possible. We also faced limitations related to data availability and documentation practices in unstructured EHRs. Several candidate predictors, including laboratory and anthropometric variables such as BMI, lipid profile, and HbA1c, exhibited very high levels of missingness (>70–95%) and were therefore excluded using a predefined threshold (>50%) to avoid unreliable imputations. This limited availability reflects routine clinical documentation in free-text hospital records and may contribute to information bias. Nevertheless, despite substantial missingness in some key variables, the model achieved moderate discriminatory performance, suggesting that unstructured EHR narratives retain meaningful clinical signal for HF risk stratification. Another methodological limitation relates to the assumption that undocumented categorical variables represent absence. In routine hospital practice, major comorbidities are typically recorded when present; therefore, the absence of a clinician-documented mention was used as an operational proxy for non-presence. This assumption may introduce information bias, as lack of documentation does not necessarily confirm true absence. However, this risk is partially mitigated by the use of longitudinal EHR data encompassing all available hospital interactions for each patient, reducing the likelihood that major comorbidities would remain entirely undocumented if truly present. In this regard, this pragmatic approach is common in real-world evidence studies and has been consistently applied within the Diabetic@ framework, including prior studies characterizing the hospital-attended diabetic population [23] and developing a CKD prediction model [19] from unstructured EHR text, both of which demonstrated clinically plausible and robust performance. Regarding predictive performance, the model exhibited high sensitivity but relative low precision. While this implies the presence of false-positive predictions, their clinical impact of such errors is limited in this context, as a positive prediction would primarily prompt closer monitoring or further clinical evaluation rather than invasive or high-risk interventions. Additionally, precision must be interpreted in light of the low prevalence of iHF in the target population, which inherently constrains achievable precision. Overall, this recall-oriented performance profile is consistent with the intended use of the model as a risk-stratification tool to identify individuals who may benefit from additional clinical evaluation rather than as a diagnostic instrument. Finally, the lack of external validation in healthcare systems outside of the participating Spanish hospitals may limit the generalizability of our findings. Future efforts should focus on validating the model across diverse settings and integrating it with complementary predictive tools. Combining HF and CKD risk prediction models developed within the Diabetic@ framework could provide a unified platform for identifying high-risk individuals across the cardio-renal spectrum, enabling more comprehensive risk stratification and supporting early, individualized intervention strategies in T2DM management.

## 5. Conclusions

The Diabetic@ project enabled us to generate an extensive, real-world database of individuals with T2DM managed in a hospital setting using unstructured data from EHRs extracted through cNLP. Building on our previous work, we developed a predictive model for two-year HF risk. The model’s combination of expert refinement, interpretability, automated prediction generation, and integration into a ready-to-use web tool (P2P) highlights its potential to support early detection, targeted monitoring, and preventive interventions in routine hospital care. Together with previous Diabetic@ contributions, this study advances the development of a scalable, multi-outcome risk stratification platform addressing the cardiovascular–renal–metabolic continuum in high-risk individuals. Future efforts should prioritize improving documentation practices, incorporating structured and primary care data, and conducting external validation studies across diverse healthcare systems to confirm real-world clinical effectiveness and expand predictive horizons.

## Figures and Tables

**Table 1:** T2DM patients used to develop the predictive model.

	Training Set			Validation Set		
	Predictive iHF	Predictive Non-iHF	Overall	Predictive iHF	Predictive Non-iHF	Overall
	(N = 43,479)	(N = 277,029)	(N = 320,508)	(N = 3756)	(N = 29,107)	(N = 32,863)
**Demographic characteristics**						
Age at index—years, median (Q1; Q3)	68 (55; 80)	54 (39; 68)	56 (41; 70)	76 (65; 84)	67 (55; 79)	68 (56; 79)
Female sex, n (%)	21,638 (49.8)	147,932 (53.4)	169,570 (52.9)	1670 (44.5)	13,764 (47.3)	15,434 (47.0)
Smoking (current/former), n (%)	13,179 (30.3)	61,295 (22.1)	74,474 (23.2)	977 (26.0)	5837 (20.1)	6814 (20.7)
**Comorbidities and T2DM-related complications, n (%) ^1^**						
Dyslipidemia	24,267 (55.8)	167,597 (60.5)	191,864 (59.9)	1964 (52.3)	15,164 (52.1)	17,128 (52.1)
Chronic kidney disease	11,768 (27.1)	34,779 (12.6)	46,547 (14.5)	1281 (34.1)	6079 (20.9)	7360 (22.4)
Ischemic heart disease	10,384 (23.9)	31,150 (11.2)	41,534 (13.0)	881 (23.5)	3608 (12.4)	4489 (13.7)
Peripheral vascular disease	7463 (17.2)	19,557 (7.1)	27,020 (8.4)	693 (18.5)	3006 (10.3)	3699 (11.3)
Atrial fibrillation	6785 (15.6)	15,111 (5.5)	21,896 (6.8)	741 (19.7)	2766 (9.5)	3507 (10.7)
Cerebrovascular disease	4546 (10.5)	10,572 (3.8)	15,118 (4.7)	341 (9.1)	1755 (6)	2096 (6.4)
Obesity	4130 (9.5)	16,557 (6.0)	20,687 (6.5)	487 (13.0)	2892 (9.9)	3379 (10.3)
Peripheral arterial disease	1936 (4.5)	4456 (1.6)	6392 (2.0)	226 (6.0)	756 (2.6)	982 (3.0)
Diabetic retinopathy	1170 (2.7)	5211 (1.9)	6381 (2.0)	203 (5.4)	848 (2.9)	1051 (3.2)
Diabetic neuropathy	555 (1.3)	2296 (0.8)	2851 (0.9)	43 (1.1)	279 (1.0)	322 (1.0)
Foot amputation	143 (0.3)	306 (0.1)	449 (0.1)	38 (1.0)	136 (0.5)	174 (0.5)
**Pharmacological treatments, n (%) ^2^**						
Any antihypertensive treatment	20,807 (47.9)	71,252 (25.7)	92,059 (28.7)	2122 (56.5)	11,098 (38.1)	13,220 (40.2)
ACE inhibitors	8483 (19.5)	31,419 (11.3)	39,902 (12.4)	730 (19.4)	3942 (13.5)	4672 (14.2)
Beta-blocking agents	7816 (18.0)	20,728 (7.5)	28,544 (8.9)	826 (22.0)	3599 (12.4)	4425 (13.5)
Angiotensin II receptor antagonists	7219 (16.6)	24,964 (9.0)	32,183 (10.0)	803 (21.4)	4288 (14.7)	5091 (15.5)
Loop diuretics	5720 (13.2)	9552 (3.4)	15,272 (4.8)	928 (24.7)	3011 (10.3)	3939 (12.0)
Calcium channel blockers	5179 (11.9)	14,681 (5.3)	19,860 (6.2)	415 (11.0)	1810 (6.2)	2225 (6.8)
Potassium-sparing agents	1643 (3.8)	3832 (1.4)	5475 (1.7)	208 (5.5)	711 (2.4)	919 (2.8)
Low-ceiling diuretics (thiazides)	1634 (3.8)	4395 (1.6)	6029 (1.9)	73 (1.9)	429 (1.5)	502 (1.5)
Low-ceiling diuretics (other)	665 (1.5)	3006 (1.1)	3671 (1.1)	85 (2.3)	467 (1.6)	552 (1.7)
Unspecified antihypertensives	1918 (4.4)	4663 (1.7)	6581 (2.1)	204 (5.4)	772 (2.7)	976 (3.0)
Statins	12,079 (27.8)	43,509 (15.7)	55,588 (17.3)	1076 (28.6)	6329 (21.7)	7405 (22.5)
Antiplatelet agents	10,506 (24.2)	30,990 (11.2)	41,496 (12.9)	995 (26.5)	5452 (18.7)	6447 (19.6)
Anticoagulants	4307 (9.9)	8686 (3.1)	12,993 (4.1)	623 (16.6)	2040 (7.0)	2663 (8.1)
Fibrates	1141 (2.6)	4973 (1.8)	6114 (1.9)	127 (3.4)	743 (2.6)	870 (2.6)
Ezetimibe	622 (1.4)	2408 (0.9)	3030 (0.9)	54 (1.4)	328 (1.1)	382 (1.2)
Bile-acid sequestrants	94 (0.2)	258 (0.1)	352 (0.1)	4 (0.1)	43 (0.1)	47 (0.1)
**Clinical parameters ^3^**						
Weight, kg						
N (%)	2939 (6.8)	12,396 (4.5)	15,335 (4.8)	147 (3.9)	896 (3.1)	1043 (3.2)
Median (Q1; Q3)	75 (62; 88)	74 (63; 86.5)	74 (62.8; 87.0)	73.5 (61.9; 87.0)	75 (63; 89)	75 (63; 89)
Height, meters						
N (%)	2111 (4.9)	8568 (3.1)	10,679 (3.3)	190 (5.1)	1543 (5.3)	1733 (5.3)
Median (Q1; Q3)	1.6 (1.6; 1.7)	1.6 (1.6; 1.7)	1.6 (1.6; 1.7)	1.6 (1.5; 1.7)	1.6 (1.6; 1.7)	1.6 (1.6; 1.7)
Body mass index, kg/m^2^						
N (%)	1180 (2.7)	4659 (1.7)	5839 (1.8)	121 (3.2)	1074 (3.7)	1195 (3.6)
Median (Q1; Q3)	28.9 (24.0; 34.4)	29.3 (24.9; 34.6)	29.1 (24.8; 34.5)	30.0 (26.0; 37.0)	30.0 (26.0; 35.0)	30.0 (26.0; 35.0)
HbA1c, %						
N (%)	2722 (6.3)	9302 (3.4)	12,024 (3.8)	815 (21.7)	4933 (16.9)	5748 (17.5)
Median (Q1; Q3)	6.2 (5.6; 7.2)	6.1 (5.6; 7.0)	6.1 (5.6; 7.1)	6.8 (6.0; 8.0)	6.7 (6.0; 7.9)	6.7 (6.0; 7.9)
GFR, mL/min/1.73 m^2^						
N (%)	19,304 (44.4)	88,332 (31.9)	10,7636 (33.6)	2259 (60.1)	15,115 (51.9)	17,374 (52.9)
Median (Q1; Q3)	73.7 (52.1–93.8)	85.3 (68.6–101.9)	83.6 (65.6–100.7)	59.4 (38.7–76.1)	60 (55–86.3)	60 (52.8–85)
Creatinine in blood, mg/dL						
N (%)	18,913 (43.5)	87,913 (31.7)	106,826 (33.3)	2265 (60.3)	15,158 (52.1)	17,423 (53)
Median (Q1; Q3)	0.9 (0.7–1.2)	0.8 (0.7–1)	0.8 (0.7–1)	1.1 (0.8–1.6)	0.9 (0.7–1.1)	0.9 (0.7–1.2)
Proteins in urine, mg/dL						
N (%)	1851 (4.3)	11,997 (4.3)	13,848 (4.3)	291 (7.7)	2078 (7.1)	2369 (7.2)
Median (Q1; Q3)	5.8 (0.1; 75)	0.2 (0.1; 2.5)	0.2 (0.1; 10)	25 (25; 75)	25 (25; 75)	25 (25; 75)
Albumin to creatinine ratio, mg/g						
N (%)	141 (0.3)	531 (0.2)	672 (0.2)	87 (2.3)	389 (1.3)	476 (1.4)
Median (Q1; Q3)	43.2 (14.9; 159)	21.6 (7.4; 80.7)	25.6 (8.7; 96.9)	47.6 (8.3; 169.9)	24.2 (4.9; 136.5)	29.1 (5.4; 145.1)
Albumin in urine, mg/24 h						
N (%)	244 (0.6)	932 (0.3)	1176 (0.4)	185 (4.9)	748 (2.6)	933 (2.8)
Median (Q1; Q3)	3.8 (2.3; 26.8)	3.5 (0.3; 4.5)	3.6 (0.2; 5.0)	7.3 (2.7; 51.3)	3.7 (0.8; 17.9)	4.2 (1.1; 24)
Total cholesterol, mg/dL						
N (%)	4800 (11.0)	20,806 (7.5)	25,606 (8.0)	1312 (34.9)	10,067 (34.6)	11,379 (34.6)
Median (Q1; Q3)	177 (145; 212)	185 (154; 218)	184 (153; 217)	164 (134.8; 196)	176 (146; 208)	174 (145; 207)
HDL, mg/dL						
N (%)	2908 (6.7)	12,970 (4.7)	15,878 (5)	933 (24.8)	6660 (22.9)	7593 (23.1)
Median (Q1; Q3)	47 (37; 58)	50 (40; 61)	49 (39; 61)	45 (36; 56)	47 (37; 58)	46 (37; 58)
LDL, mg/dL						
N (%)	3549 (8.2)	16,518 (6)	20,067 (6.3)	962 (25.6)	6788 (23.3)	7750 (23.6)
Median (Q1; Q3)	101 (78; 130)	107 (85; 135)	106 (84; 134)	94 (71; 122)	103 (79; 130.1)	102 (78; 129.4)
Triglycerides, mg/dL						
N (%)	4847 (11.1)	19,943 (7.2)	24,790 (7.7)	1289 (34.3)	8875 (30.5)	10,164 (30.9)
Median (Q1; Q3)	118 (85; 170)	113 (80; 165)	114 (81; 166)	115 (84; 163)	111 (80; 158)	112 (80; 158)

Abbreviations: GFR, glomerular filtration rate; HbA1c, glycated hemoglobin; HDL, high-density lipoprotein; iHF: incident heart failure; LDL, low-density lipoprotein; non-iHF: non-incident heart failure; T2DM, type 2 diabetes mellitus. ^1^ The presence of each feature was analyzed at index with a window of (−Inf, 0] months. ^2^ The presence of each feature was analyzed at index with a window of (−12, 0] months. ^3^ The closest occurrence of each feature was analyzed at index with a window of (−12, 0] months. Note: Bold formatting and background shading are used to highlight section subheadings that group related variables within the table.

**Table 2:** Performance of machine learning prediction models.

Model	AUC-ROC	Accuracy	Precision	Recall	F1-Score	F2-Score
**Cross-validation metrics of the regularized models, mean (CI)**						
**Full models**						
XGB Classifier	0.74 (0.73–0.75)	0.68 (0.63–0.74)	0.26 (0.24–0.28)	0.67 (0.61–0.73)	0.37 (0.35–0.39)	0.5 (0.48–0.52)
Logistic Regression	0.73 (0.72–0.75)	0.69 (0.64–0.74)	0.26 (0.24–0.28)	0.65 (0.59–0.71)	0.37 (0.35–0.39)	0.49 (0.47–0.51)
Random Forest Classifier	0.57 (0.56–0.57)	0.78 (0.76–0.8)	0.22 (0.21–0.23)	0.25 (0.22–0.27)	0.23 (0.22–0.24)	0.24 (0.23–0.25)
Decision Tree Classifier	0.51 (0.51–0.52)	0.74 (0.72–0.77)	0.22 (0.21–0.23)	0.34 (0.31–0.37)	0.26 (0.26–0.27)	0.3 (0.29–0.32)
**Reduced and refined models**						
Logistic regression reduced	0.73 (0.71–0.75)	0.69 (0.64–0.74)	0.26 (0.24–0.28)	0.64 (0.57–0.71)	0.37 (0.35–0.39)	0.49 (0.47–0.51)
Logistic regression refined	0.73 (0.71–0.75)	0.69 (0.64–0.74)	0.26 (0.24–0.28)	0.65 (0.58–0.72)	0.37 (0.35–0.39)	0.49 (0.47–0.51)
**Validation metrics of the logistic regression models, mean (95% CI)**						
Full model	0.69 (0.68–0.70)	0.51 (0.51–0.52)	0.16 (0.16–0.17)	0.79 (0.78–0.80)	0.27 (0.26–0.27)	0.44 (0.44–0.45)
Reduced model	0.68 (0.68–0.69)	0.51 (0.51–0.52)	0.16 (0.16–0.16)	0.78 (0.77–0.79)	0.27 (0.26–0.27)	0.44 (0.43–0.45)
Refined model	0.68 (0.68–0.69)	0.51 (0.51–0.52)	0.16 (0.16–0.16)	0.77 (0.76–0.79)	0.26 (0.26–0.27)	0.44 (0.43–0.45)

Abbreviations: CI, confidence interval; AUC-ROC, area under the curve of the receiver operating characteristic; XGB, eXtreme Gradient Boosting. Note: Bold formatting and background shading are used to highlight section subheadings.

## Data Availability

The data underlying this article was provided to the authors by permission of the affiliate institutions. Data could be shared on reasonable request to the corresponding author, after permission of the institutions involved.

## Supplementary Materials

The following supporting information can be downloaded at: https://www.mdpi.com/article/10.3390/jcm15093287/s1, Figure S1: Receiving Operating Characteristic (ROC) curve for the performance of the logistic regression model; Figure S2: Cumulative incidence of major cardiovascular comorbidities and complications in persons with T2DM and pHF; Figure S3: Evolution of laboratory variables in persons with T2DM and pHF; Table S1: Participating centers by region; Table S2: Reading performance of EHRead^®^ Technology; Table S3: List of potential predictors; Table S4: Patient demographic, clinical and analytical characteristics at baseline of persons with T2DM; Table S5: Variable importance in the Logistic Regression models.
