# Supplementary material for: Natural Language Processing of Unstructured Healthcare Data for Predicting Heart Failure in Individuals with Type 2 Diabetes
Source: J Clin Med. 2026 Apr 25;15(9):3287. doi: 10.3390/jcm15093287 (PMC13164468; doi:10.3390/jcm15093287)
Supplement: Supplementary file 1 [file jcm-15-03287-s001.zip › jcm-4226393-supplementary.pdf]

SUPPLEMENTARY MATERIAL

Natural Language Processing of Unstructured Healthcare Data for Predicting Heart Failure in Individuals with Type 2 Diabetes

Table of Contents

1. Supplementary Methods 3

1.1. Predictive Model Development and Validation 3

2. Supplementary Results 5

2.1. Descriptive analysis 5

2.2. Supplementary Tables 7

2.3. Supplementary Figures 16

Supplemental Tables

Table S1. Participating centers by region 7

Table S2. Reading performance of EHRead® Technology 8

Table S3. List of potential predictors 9

Table S4. Patient demographic, clinical and analytical characteristics at baseline of persons with T2DM 11

Table S5. Variable importance in the Logistic Regression models 14

Supplemental Figures

Figure S1. Receiving Operating Characteristic (ROC) curve for the performance of the logistic regression model. 16

Figure S2. Cumulative incidence of major cardiovascular comorbidities and complications in persons with T2DM and pHF. 17

Figure S3. Evolution of laboratory variables in persons with T2DM and pHF 19

1. Supplementary Methods

1.1. Predictive Model Development and Validation

The HF predictive model included patients from six hospitals in Madrid, Catalonia, Valencia, and the Balearic Islands as the training set (n = 320,508 patients, 90%), and two hospitals in Castilla y León as the validation set (n = 32,863; 10%). Both sets were divided into positive (incident HF [iHF] within 2 years) and negative classes (non-iHF). Forty-two potential predictors, including demographics, comorbidities, and treatments, were identified by medical experts (Table S3). Variables with over 50% missing data were excluded.

We trained four ML-based predictive models: decision tree (DT), random forest (RF), extreme gradient boosting (XGB), and logistic regression (LR), by fitting the classifier algorithms to the training set, and to ensure reproducibility, we used the default hyperparameter settings and fixed a random seed. To address potential class imbalance-related issues, we trained the models with regularization, balancing the weights for each class (i.e., the positive class was given a weight equivalent to the ratio of the frequencies of the negative/positive courses).

Model performance was assessed using stratified 10-fold cross-validation on the training set, as measured by the area under the curve of the receiver operating characteristic (AUC-ROC), as well as precision (positive predictive value), recall (sensitivity), accuracy, F1-score, and F2-score. Confidence intervals (CIs) were estimated using the t-interval method, based on the mean and standard error of the metrics obtained in each cross-validation fold. We select the best-performing model (i.e., one with better metrics and/or interpretability) and perform feature selection using the Maximum Relevance Minimum Redundancy (MRMR) method. Neither stepwise selection nor predictor regularization was employed. MRMR method would exclude variables that offer redundant information, as well as those with low predictive performance, resulting in a reduced model reviewed by expert physicians. The final models were validated

on the validation set with performance measured using ROC and precision-recall curves, and CIs estimated through bootstrapping (1,000 splits).

2. Supplementary Results

2.1. Descriptive analysis

The demographic and clinical characteristics, as well as pharmacological treatments and laboratory parameters of the patients with T2DM are described in Table S4. The median (Q1, Q3) age was 71.0 (56, 83) years in pHF patients and 56 (41, 71) years in non-pHF patients. There were slightly more females than males in both groups (50.3% and 52.3%, respectively). High blood pressure, dyslipidemia, and chronic kidney disease (CKD) were the most common comorbidities in both groups, frequencies of 67.9%, 62.2%, and 39.0% in the pHF group, and 64.7%, 59.1%, and 16.4% in the non-pHF group.

At inclusion, data on antihypertensive treatment were available for 34.2% of patients—65.8% in the pHF group and 29.0% in the non-pHF group. Among pHF individuals, beta-blockers (32.0%) and ACE inhibitors (28.5%) were most common, whereas among non-pHF patients, ACE inhibitors (12.3%) and angiotensin II receptor blockers (ARBs) (10.2%) predominated. Statin use was reported in 35.3% of pHF patients compared with 17.4% in the non-pHF group. For antidiabetic treatment, 13.8% of patients with pHF and 9.7% of patients without pHF reported using oral medications, while 7.5% of patients with pHF and 3.2% of patients without pHF used insulin or insulin analogs.

Regarding laboratory parameters, HbA1c levels were available for 5.8% of individuals with T2DM, including 13.5% of pHF and 4.5% of non-pHF. Median (Q1, Q3) HbA1c values for these groups were 6.1% (5.6; 7.0) and 6.2% (5.6; 7.3), respectively. Total cholesterol levels were reported in 23.8% of patients in the pHF group and 9.9% in the non-pHF group, with median (Q1, Q3) values of 163 (134; 197) mg/dL and 180 mg/dL (149; 213) mg/dL, respectively.

Follow-up data were available for 85.5% of the individuals for ≥12 months and 35.1% for ≥48 months. The median (Q1, Q3) follow-up duration was 38.2 (20.3; 54.0) months. Figure S2 depicts the cumulative incidence of significant comorbidities and complications in the pHF group, including CKD, Ischemic Heart Disease (IHD), Atrial Fibrillation (AF), and Cerebrovascular Disease (CBVD). Notably, the cumulative incidence at 60 months was 40% for CKD, 25% for IHD, 20% for AF, and 18% for CBVD in the pHF group. In contrast, in the non-pHF group, these complications were reported in 23% of patients for CKD, 17% for IHD, 12% for AF, and 7% for CBVD.

Figure S3 shows Smooth Plots of HbA1c, total cholesterol, and LDL cholesterol values obtained from laboratory data recorded in the unstructured dataset throughout the follow-up period. These parameters were available in less than 20% of the individuals involved in the study. In the pHF group, HbA1c levels were lower and tended to decrease over time. In contrast, the non-pHF group showed a slight increase in HbA1c levels by the end of the follow-up. Both total cholesterol and LDL cholesterol levels progressively decreased in both groups; however, the pHF group consistently maintained lower levels throughout the follow-up period.

2.2. Supplementary Tables

Table S1. Participating centers by region

Center	Region
Hospital Universitario de Fuenlabrada	Madrid
Hospital Universitario Puerta de Hierro	Madrid
Hospital Universitario Infanta Sofía	Madrid
Hospital de la Santa Creu i Sant Pau	Catalonia
Complejo Asistencial Universitario de León	Castilla y León
Hospital Universitario Río Hortega de Valladolid	Castilla y León
Hospital Universitari i Politècnic La Fe	Valencia
Hospital Son Espases	Balearic Islands

Table S2. Reading performance of EHRead® Technology

Variable	Recall	Precision	F1-score	IAA F1-score
Saxagliptin	0.936	1.000	0.967	0.960
Obesity	0.931	0.955	0.943	0.934
Insulin glargine	0.867	0.993	0.926	0.932
Hypertriglyceridemia	0.860	0.945	0.901	0.923
Insulin aspartate	0.896	0.885	0.890	0.890
HbA1c	0.826	0.934	0.877	0.916
HDL	0.781	0.996	0.875	0.932
T2DM	0.799	0.961	0.873	0.930
Diabetic retinopathy	0.822	0.910	0.864	0.892
Insulin lispro protamine	0.691	0.985	0.812	0.955
Foot amputation	0.667	0.914	0.771	0.846
T1DM	0.724	0.808	0.764	0.930
Hypercholesterolemia	0.593	0.973	0.737	0.897
Diabetic nephropathy	0.960	0.440	0.603	0.932
Glomerular filtration rate	0.429	0.947	0.590	0.855

Abbreviations: HbA1c, glycated hemoglobin; HDL, high-density lipoprotein; IAA, inter-annotator agreement; T1DM, type 1 diabetes mellitus; T2DM, type 2 diabetes mellitus.

Table S3. List of potential predictors

Type	Variable	Definition	Look-back window
Patient characteristics	Female sex	Binary	NA
	Age at index (years)	Numeric	0
	Smoking	Binary	Infinite
	Obesity	Binary	Infinite
	Chronic kidney disease	Binary	Infinite
	Peripheral vascular disease	Binary	Infinite
	Atrial fibrillation	Binary	Infinite
	Ischemic heart disease	Binary	Infinite
	Cerebrovascular disease	Binary	Infinite
	Peripheral arterial disease	Binary	Infinite
	Diabetic neuropathy	Binary	Infinite
	Diabetic retinopathy	Binary	Infinite
	Foot amputation	Binary	Infinite
Measurable parameters	Weight measurement	Numeric	1 year
	Height measurement	Numeric	1 year
	BMI measurement	Numeric	1 year
	SBP measurement	Numeric	1 year
	DBP measurement	Numeric	1 year
	HbA1c measurement	Numeric	1 year
	GFR measurement	Numeric	1 year
	Creatinine in blood measurement	Numeric	1 year
	Proteins in urine measurement	Numeric	1 year
	Albumin to creatinine ratio measurement	Numeric	1 year
	Albumin in urine measurement	Numeric	1 year
	Total cholesterol measurement	Numeric	1 year
	HDL measurement	Numeric	1 year
	LDL measurement	Numeric	1 year
	Triglycerides measurement	Numeric	1 year

Treatments	Antiplatelet agents	Binary	1 year
	Anticoagulants	Binary	1 year
	Beta-blocking agents	Binary	1 year
	ACE inhibitors	Binary	1 year
	Angiotensin II receptor antagonists	Binary	1 year
	Loop diuretics	Binary	1 year
	Low-ceiling diuretics (thiazides)	Binary	1 year
	Low-ceiling diuretics (other)	Binary	1 year
	Potassium-sparing agents	Binary	1 year
	Statins	Binary	1 year
	Ezetimibe	Binary	1 year
	Fibrates	Binary	1 year
	Bile-acid sequestrants	Binary	1 year
	Calcium channel blockers	Binary	1 year

Abbreviations: ACE, angiotensin-converting enzyme; BMI, body mass index; DBP, diastolic blood pressure; GFR, glomerular filtration rate; HbA1c, glycated hemoglobin; HDL, high-density lipoprotein cholesterol; HF, heart failure; Inf, infinite; LDL, low-density lipoprotein cholesterol NA, not applicable; SBP, systolic blood pressure.

Table S4. Patient demographic, clinical and analytical characteristics at baseline of persons with T2DM

	T2DM		
	**pHF (N = 84,197)**	**non-pHF (N = 504,559)**	**overall (N = 588,756)**
**Demographic characteristics**			
Age at index - years			
Available (%)	84,197 (100.0)	504,559 (100.0)	588,756 (100.0)
Median (Q1, Q3)	71 (56, 83)	56 (41, 71)	59 (43, 73)
≥ 65 years, n (%)	52,633 (62.5)	181,486 (36.0)	234,119 (39.8)
Female sex, n (%)	42,341 (50.3)	263,994 (52.3)	306,335 (52.0)
Body mass index, kg/m2			
Available, n (%)	2,791 (3.3)	9,520 (1.9)	12,311 (2.1)
Median (Q1; Q3)	28.4 (23.9; 34.3)	29.1 (24.8; 34.7)	29.0 (24.5; 34.6)
Systolic blood pressure, mmHg			
Available, n (%)	39,029 (46.4)	161,203 (31.9)	200,232 (34.0)
Median (Q1; Q3)	131.0 (116; 150)	135 (120; 151)	135 (120; 150)
Diastolic blood pressure, mmHg			
Available, n (%)	39,054 (46.4)	161,339 (32.0)	200,393 (34.0)
Median (Q1; Q3)	74 (64; 84)	78 (69; 87)	77 (68; 86)
Smoking history (current or former), n (%)	28,757 (34.2)	115,055 (22.8)	143,812 (24.4)
**Comorbidities, n (%) ***			
High blood pressure	57,132 (67.9)	326,209 (64.7)	383,341 (65.1)
Dyslipidemia	52,388 (62.2)	298,186 (59.1)	350,574 (59.5)
Chronic kidney disease	32,870 (39.0)	82,943 (16.4)	115,813 (19.7)
Ischemic heart disease	31,871 (37.9)	63,759 (12.6)	95,630 (16.2)
Atrial fibrillation	25,531 (30.3)	35,027 (6.9)	60,558 (10.3)
Obesity **	12,081 (14.3)	33,350 (6.6)	45,431 (7.7)
Cerebrovascular disease	13,655 (16.2)	23,703 (4.7)	37,358 (6.3)
Peripheral vascular disease	5,071 (6)	9,804 (1.9)	14,875 (2.5)
**Treatments, n (%)***			
Antihypertensives	55,442 (65.8)	146,105 (29.0)	2015,47 (34.2)
ACE inhibitors	24,035 (28.5)	61,900 (12.3)	85,935 (14.6)

Beta-blocking agents	26,907 (32.0)	45,080 (8.9)	71,987 (12.2)
Angiotensin II receptor blockers	16,601 (19.7)	51,265 (10.2)	67,866 (11.5)
Loop diuretics	31,022 (36.8)	25,741 (5.1)	56,763 (9.6)
Statins	29,708 (35.3)	87,590 (17.4)	117,298 (19.9)
Oral antidiabetic drugs	11,623 (13.8)	48,827 (9.7)	60,450 (10.3)
Insulins and analogs	6,346 (7.5)	16,344 (3.2)	22,690 (3.9)
**Laboratory parameters #**			
HbA1c [%]			
Available, n (%)	11,372 (13.5)	22,924 (4.5)	34,296 (5.8)
Median (Q1;Q3)	6.1 (5.6; 7.0)	6.2 (5.6; 7.3)	6.2 (5.6; 7.2)
eGFR [mL/min/1.73m2] †			
Available, n (%)	52,686 (62.6)	1838,47 (36.4)	236,533 (40.2)
Median (Q1; Q3)	68.2 (48.0; 90.2)	82.4 (62.2; 100.0)	79.9 (60.0; 98.3)
Proteins in urine (mg/dL)			
Available, n (%)	6,285 (7.5)	21,908 (4.3)	28,193 (4.8)
Median (Q1; Q3)	0.8 (0.1; 50.0)	0.3 (0.1; 30.0)	0.5 (0.1; 30.0)
Total cholesterol (mg/dL)			
Available, n (%)	20,075 (23.8)	49,806 (9.9)	69,881 (11.9)
Median (Q1; Q3)	163 (134; 197)	180 (149; 213)	175 (144; 209)
HDL (mg/dL)			
Available, n (%)	13,685 (16.3)	30,836 (6.1)	44,521 (7.6)
Median (Q1; Q3)	44 (36; 55)	48 (38; 60)	47 (38; 59)
LDL (mg/dL)			
Available, n (%)	16,051 (19.1)	37,015 (7.3)	53,066 (9.0)
Median (Q1; Q3)	93.0 (71.0; 119.0)	104.3 (81.6; 133.0)	101 (78.0; 129.0)
Triglycerides (mg/dl)			
Available, n (%)	19,815 (23.5)	46,757 (9.3)	66,572 (11.3)
Median (Q1; Q3)	110 (80; 157)	112 (80; 162)	111 (80; 161)

Abbreviations: ACE, angiotensin converting enzyme inhibitors; eGFR, estimated glomerular filtration rate; HbA1c, glycated hemoglobin; HDL, high-density lipoprotein; HF, heart failure; IQR, interquartile range; LDL, Low-density lipoprotein; nonpHF, nonprevalent heart failure; pHF, prevalent heart failure; SD, standard deviation; T2DM, type 2 diabetes mellitus.
*Data extracted and analyzed considering all information up to inclusion date.
**Obesity was extracted as both direct variable (e.g., mention of obesity in EHRs) and inferred variable (i.e., BMI ≥30).
#Data extracted and analyzed considering all information between 12 months before inclusion date.

†eGFR data were extracted as both direct variable (i.e., mention of eGFR value in EHRs) and inferred variable (i.e., a posteriori calculation using CKD-EPI creatinine equation, with available patient information, including sex, age, and creatinine).

Table S5. Variable importance in the Logistic Regression models

	Full model (27 predictors)		Reduced model (12 predictors)		Refined model (9 predictors)	
**Feature**	**β-coefficient**	**Odds Ratio**	**β-coefficient**	**Odds Ratio**	**β-coefficient**	**Odds Ratio**
Chronic kidney disease	0.571	1.770	0.582	1.790	0.575	1.778
Loop diuretics	0.503	1.653	0.558	1.747	0.572	1.773
Diabetic neuropathy	0.460	1.585	—	—	—	—
Atrial fibrillation	0.425	1.530	0.454	1.574	0.530	1.698
Cerebrovascular disease	0.394	1.483	0.404	1.498	0.435	1.545
Ischemic heart disease	0.393	1.481	0.392	1.481	0.446	1.562
Peripheral vascular disease	0.353	1.423	0.377	1.459	0.392	1.481
Smoking (current/former)	0.350	1.420	0.367	1.444	0.371	1.449
Potassium-sparing agents	0.337	1.401	—	—	—	—
Obesity	0.291	1.337	—	—	—	—
Foot amputation	0.235	1.265	—	—	—	—
Anticoagulants	0.215	1.239	0.204	1.226	—	—
Low-ceiling diuretics (thiazides)	0.208	1.232	—	—	—	—
Bile acid sequestrants	0.177	1.194	—	—	—	—
Antihypertensives	0.164	1.178	0.110	1.116	0.184	1.202
Beta blocking agents	0.138	1.149	0.169	1.184	—	—
Antiplatelet agents	0.109	1.115	0.106	1.111	—	—

Calcium channel blockers	0.101	1.106	—	—	—	—
Peripheral arterial disease	0.049	1.050	—	—	—	—
Fibrates	0.034	1.034	—	—	—	—
Age at index	0.027	1.028	0.027	1.027	0.027	1.027
Statins	–0.040	0.960	—	—	—	—
Female sex	–0.058	0.944	—	—	—	—
Diabetic retinopathy	–0.066	0.937	—	—	—	—
Angiotensin II receptor antagonists	–0.088	0.916	—	—	—	—
Angiotensin-converting enzyme inhibitors	–0.124	0.883	—	—	—	—
Low-ceiling diuretics (other)	–0.208	0.812	—	—	—	—

2.3. Supplementary Figures

Figure S1. Receiving Operating Characteristic (ROC) curve for the performance of the logistic regression model.

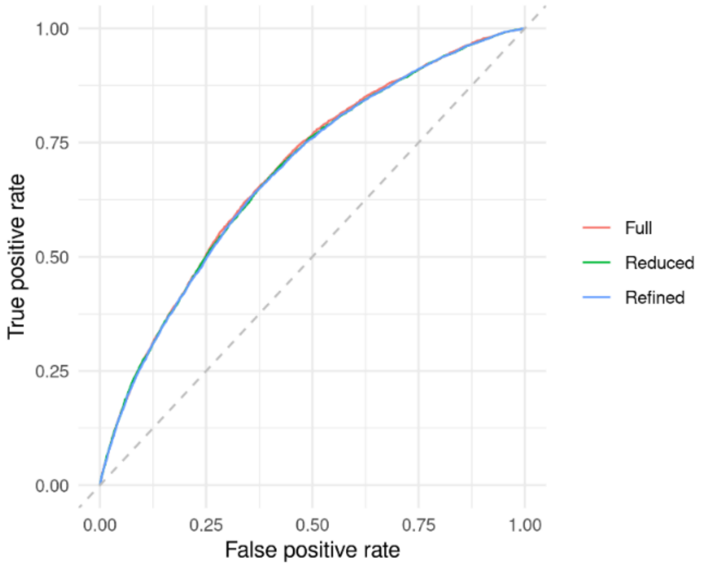

Performance of the Logistic Regression predictive model		
	Cross-validation ROC-AUC	Validation ROC-AUC
**Full (mean, 95% CI)**	0.73 (0.72-0.75)	0.69 (0.68-0.70)
**Reduced (mean, 95% CI)**	0.73 (0.71-0.75)	0.68 (0.68-0.69)
**Refined (mean, 95% CI)**	0.73 (0.72-0.75)	0.68 (0.68-0.69)

Figure S2. Cumulative incidence of major cardiovascular comorbidities and complications in persons with T2DM and pHF.

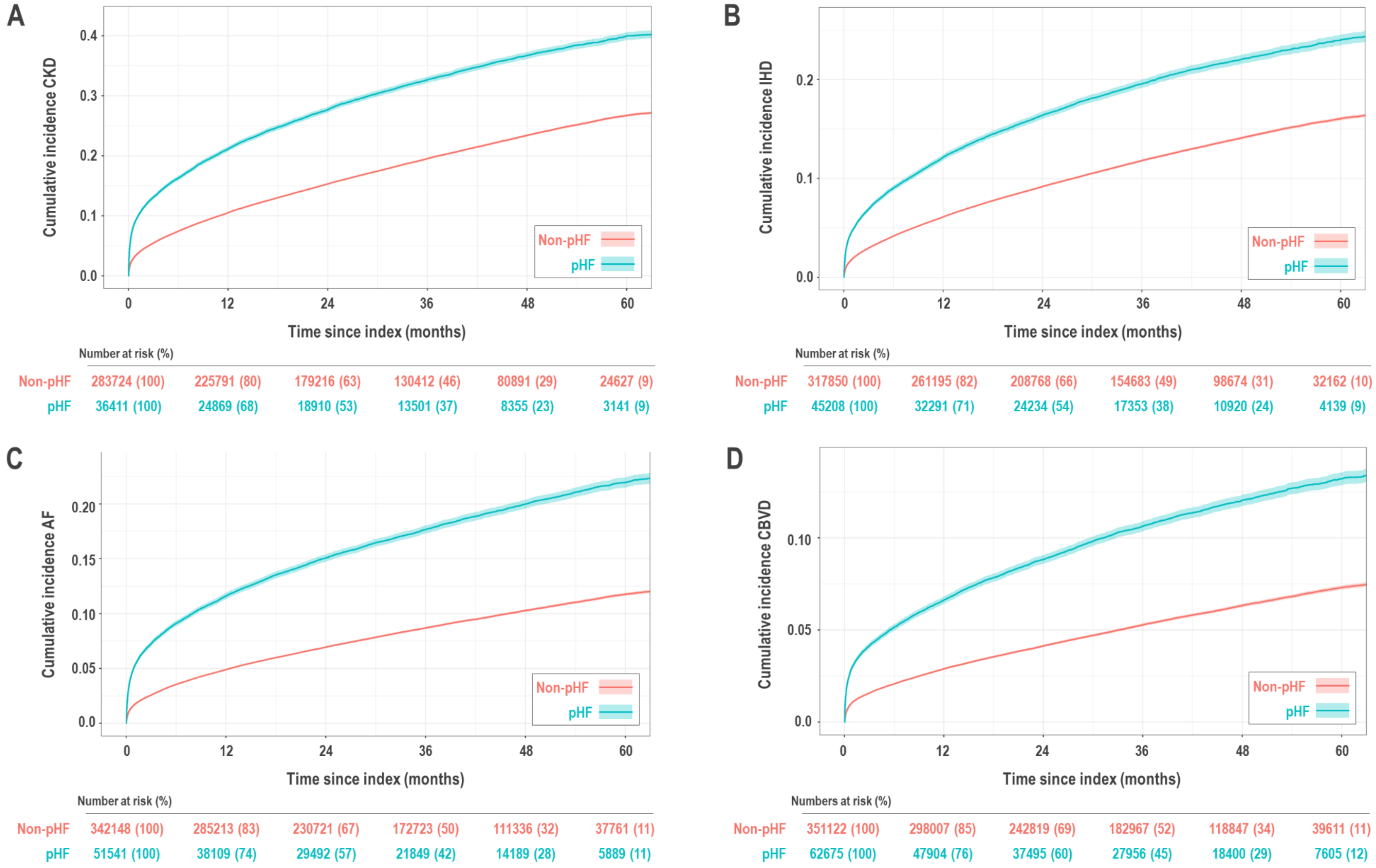

Cumulative incidence of major cardiovascular comorbidities and complications in persons with T2DM and pHF, *including CKD (panel A), IHD (panel B), AF (panel C), and CBVD (panel D). Abbreviations: CKD, chronic kidney disease; IDH ischemic heart disease; AF, atrial fibrillation; CBVD, cerebrovascular disease; non-pHF, non-prevalent heart failure; pHF, prevalent heart failure.*

Figure S3. Evolution of laboratory variables in persons with T2DM and pHF

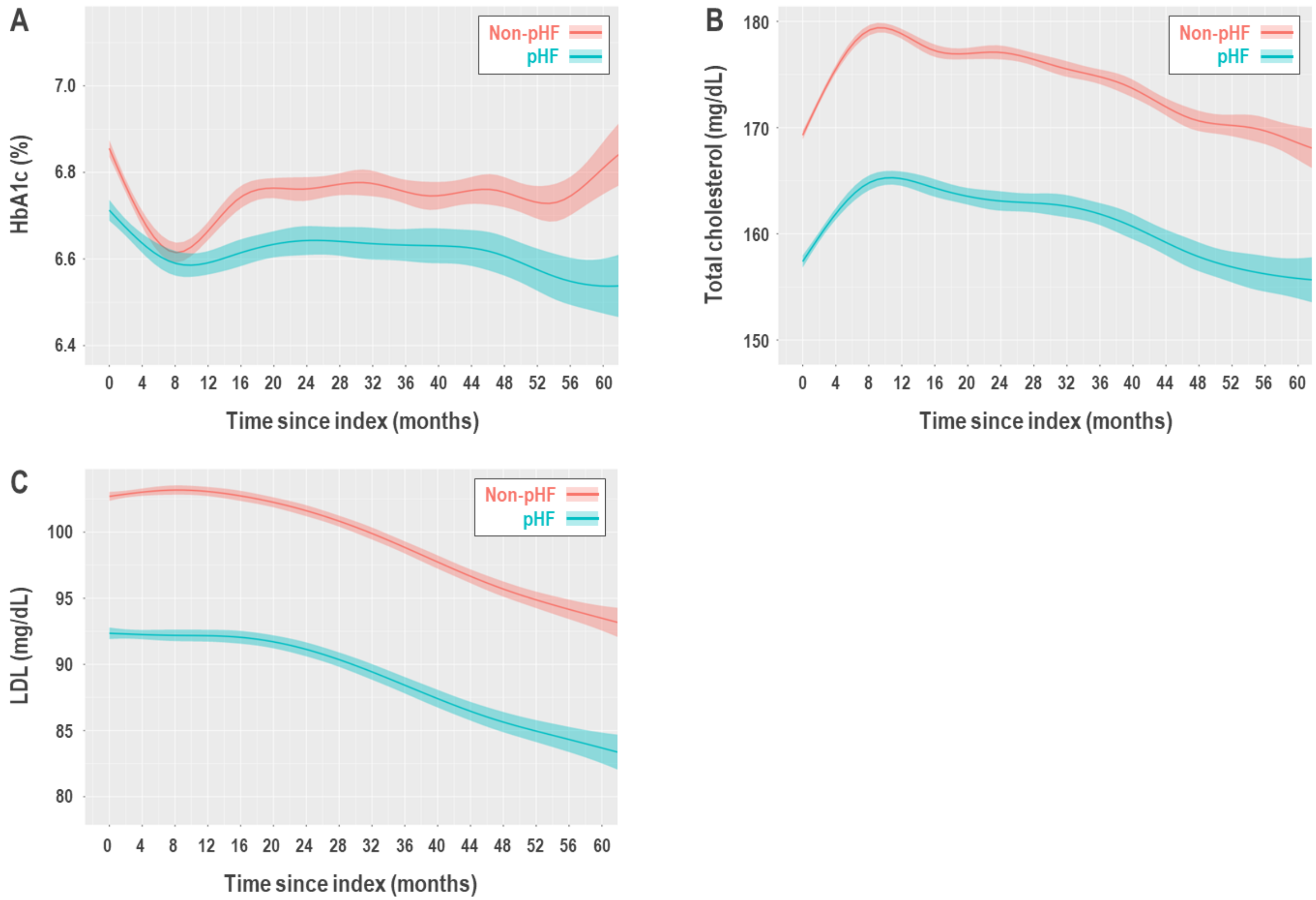

Abbreviations: HbA1c, glycated hemoglobin; LDL, Low-Density Lipoprotein; non-pHF, non-prevalent heart failure; pHF, prevalent heart failure.
